# Testing an Iron Oxide Nanoparticle-Based Method for Magnetic Separation of Nanoplastics and Microplastics from Water

**DOI:** 10.3390/nano12142348

**Published:** 2022-07-09

**Authors:** Leisha M. A. Martin, Jian Sheng, Paul V. Zimba, Lin Zhu, Oluniyi O. Fadare, Carol Haley, Meichen Wang, Timothy D. Phillips, Jeremy Conkle, Wei Xu

**Affiliations:** 1 Department of Life Sciences, Texas A&M University, Corpus Christi, TX 78412, USA; leisha.martin@tamucc.edu; 2 School of Engineering, Texas A&M University, Corpus Christi, TX 78412, USA; jian.sheng@tamucc.edu; 3 Center for Coastal Studies, Texas A&M University, Corpus Christi, TX 78412, USA; pvzimba1@gmail.com; 4 Irma Lerma Rangel College of Pharmacy, Texas A&M University, College Station, TX 77843, USA; lzhu@tamu.edu; 5 Department of Physical & Environmental Sciences, Texas A&M University, Corpus Christi, TX 78412, USA; oluniyi.fadare@tamucc.edu (O.O.F.); carol.haley@tamucc.edu (C.H.); jeremy.conkle@tamucc.edu (J.C.); 6 College of Veterinary Medicine and Biomedical Sciences, Veterinary Integrative Biosciences, Texas A&M University, College Station, TX 77843, USA; mwang@cvm.tamu.edu (M.W.); tphillips@cvm.tamu.edu (T.D.P.)

**Keywords:** iron oxide nanoparticles, hydrophobicity, hydrophobic coatings, separation science, interparticle interactions, nanoplastics, microplastics, plastic pollution, water remediation, amphiphilic polymer, PDMS, polydimethylsiloxane nanocomposite, aminopropylsiloxane

## Abstract

Nanoplastic pollution is increasing worldwide and poses a threat to humans, animals, and ecological systems. High-throughput, reliable methods for the isolation and separation of NMPs from drinking water, wastewater, or environmental bodies of water are of interest. We investigated iron oxide nanoparticles (IONPs) with hydrophobic coatings to magnetize plastic particulate waste for removal. We produced and tested IONPs synthesized using air-free conditions and in atmospheric air, coated with several polydimethylsiloxane (PDMS)-based hydrophobic coatings. Particles were characterized with scanning electron microscopy (SEM), transmission electron microscopy (TEM), superconducting quantum interference device (SQUID) magnetometry, dynamic light scattering (DLS), X-ray diffraction (XRD) and zeta potential. The IONPs synthesized in air contained a higher percentage of the magnetic spinel phase and stronger magnetization. Binding and recovery of NMPs from both salt and freshwater samples was demonstrated. Specifically, we were able to remove 100% of particles in a range of sizes, from 2–5 mm, and nearly 90% of nanoplastic particles with a size range from 100 nm to 1000 nm using a simple 2-inch permanent NdFeB magnet. Magnetization of NMPs using IONPs is a viable method for separation from water samples for quantification, characterization, and purification and remediation of water.

## 1. Introduction

Plastic materials production surpasses the production of all other synthetic materials worldwide. Of the more than 8 billion metric tons of plastic produced from 2017 to date, approximately 9% has been recycled, 12% has been incinerated, and 79% has accumulated in landfills or been released in the environment [1]. Improperly discarded plastics accumulate in the environment, where they are fragmented over time by environmental weathering, leaving nanoplastic and microplastic/particles (NMPs) behind [2]. Environmental plastic fragments are referred to as either primary or secondary NMPs. Primary NMPs are industrially produced and introduced to the environment already in a micro- or nano-scale state (plastic dust, microbeads, pre-production plastic pellets (nurdles), and engineered polymeric nanoparticles). The contribution of microbeads to total environmental micro- and nano-plastic pollution is low, comprising only about 10% [3,4]. Secondary NMPs, not to be confused with secondary (recycled) plastics, result from environmental degradation of bulk plastic waste. Although most of the plastic waste comes from secondary plastics, primary plastics remain contaminants of concern due to their small sizes [3]. These plastic particles are present in drinking water, fresh and salt water, air, and soil sediments. Prevailing data suggest that human and animal exposure to ever-accumulating NMPs will have negative consequences [5]. Others suggest that NMPs pose significant health risks to marine organisms and humans [6].

Although there are several methods employed for the removal of NMPs from water [7], at least some of these methods are limited by minimum particle size and destructive methods are not useful for laboratory particle analysis. While purification and isolation methods (filtration, evaporation, solvent extraction, density separation, or gravitational separation) exist, due to the unique properties of nanoplastics, each of these methods has shortfalls. Membrane filtration is adequate for smaller volumes, however, the flow rate through a nano-porous membrane is exceptionally slow and clogging is common. Additionally, plastic particles may remain adhered to the filters [8], reducing the detected abundance. Density separation using saturated salt solutions has been successfully employed but is not widely used due to difficulty collecting NPs from the liquid-air interface [9]. Due to difficulties in detecting and removing smaller particles, previous reports may have significantly underestimated their abundance [10].

Adsorption methods are broadly utilized to remove a variety of pollutants from waters. Adsorption is a well-characterized equilibrium separation procedure with several benefits such as cost, ease of incorporation and simplicity [11,12] Previous published works have investigated the use of adsorbents for the removal of methylene blue from solution [13]. Polydopamine microspheres have also been evaluated as high-efficiency adsorbents for the separation of organic dyes, exhibiting selective adsorption of cationic dyes [14]. Functionalized magnetic nanoparticles, specifically, have also been used to remove both cationic and anionic dyes from aqueous solutions and appear superior to other adsorbents considering ease of use and environmental impact [15].

This system of specific magnetization could address the shortfalls of current laboratory methods of NMP collection, as well as the remaining issues of wastewater treatment and environmental remediation. As products of weathering of iron-bearing minerals and biominerals, iron-based nanoparticles (NPs) occur naturally in the environment, and are found in sediments, natural water sources, rocks and minerals and volcanic ash [16]. Engineered NPs, such as iron NPs, are commonly added to the environment for ground water remediation [17,18,19], and the fate, transport, and chemical behavior of IONPs in environmental systems has been previously reviewed [16].

Incorporating EPA green chemistry methods, we produced iron oxide nanoparticles (IONPs) with different hydrophobic coatings, to separate, concentrate, and remove NMP particles from water via magnetic separation. Using this nanotechnology-based system, engineered with a focus on the properties of hydrophobicity and magnetism, we can bind, separate, isolate, and quantify micro- and nano-plastic particles from both fresh and saltwater.

## 2. Materials and Methods

### 2.1. Chemicals and Reagents

Iron (III) chloride (anhydrous), oleic acid (90%), poly(acrylic acid), sodium dodecyl sulfate (SDS), fluorescent and non-fluorescent latex beads, and heptane were purchased from Sigma Aldrich (St. Louis, MO, USA); sodium oleate was purchased from TCI America Inc. (Portland, OR, USA); Siliclad^®^ and monocarboxydecyl terminated polydimethylsiloxane, 2–3% aminopropylmethyl siloxane-dimethylsiloxane co-polymer, 4–5% aminopropylmethyl siloxane-dimethylsiloxane co-polymer (PDMS-co-APMS), and Siliclad^®^, were purchased from Gelest Inc. (Morrisville, PA, USA); 1-octadecene was purchased from EMD Millipore (Burlington, MA, USA); ethanol, acetone were purchased from Fisher Scientific Co. (Hampton, NH, USA); hydroxy terminated poly (dimethylsiloxane) (4.2 k) was purchased from Alfa Aesar (Ward Hill, MA, USA); polyethylene fiber (30 μm LDPE) was purchased from Lumat Group (Richmond, VA, USA). All chemicals were used as received without further purification.

### 2.2. Synthesis of IONPs

IONPs were produced using a modified solvothermal procedure based on the thermal decomposition of iron oleate [20]. Green chemistry methods and modifications include the use of soluble iron chloride salts and the use of heptane instead of hexane. We found that larger particles could increase the reagent/solvent ratio threefold. However, this modification also increases polydispersity. IONPs were synthesized using air-free procedures (under argon) or in ambient air for comparison.

### 2.3. Capping Procedure

**Figure 1:**
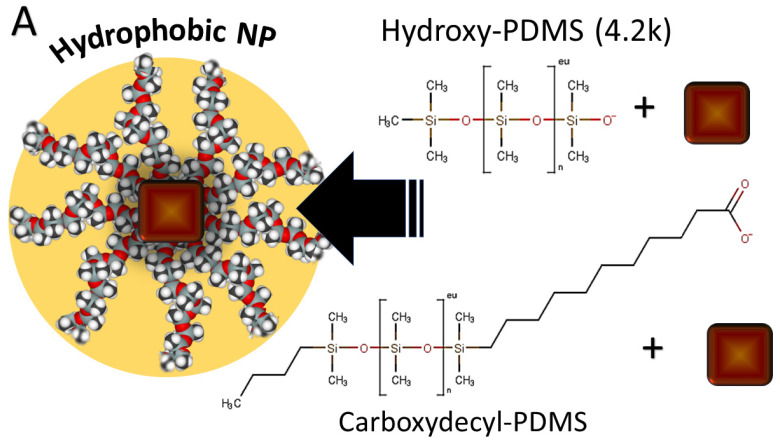

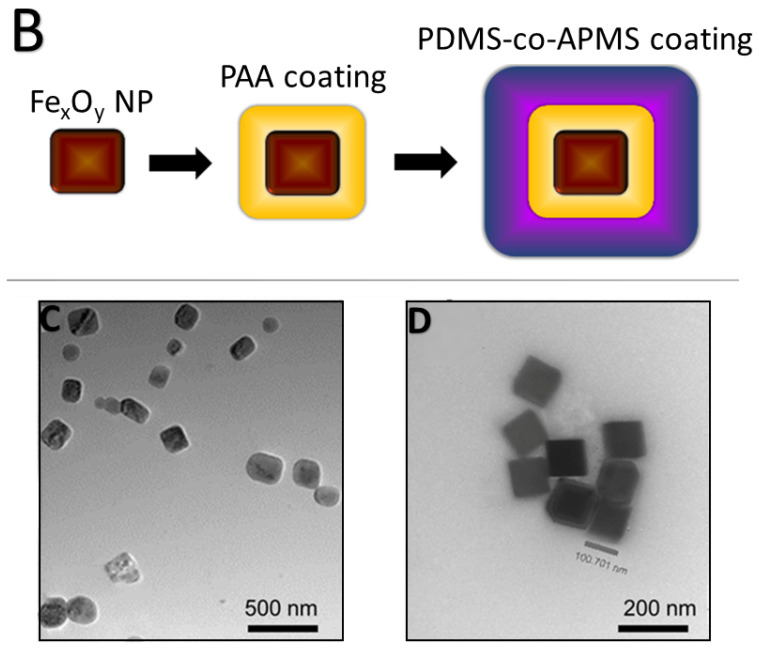
(**A**) Functionalization of IONPs with PDMS via hydroxy-terminated PDMS (PDMS-OH, upper structure) or carboxy-terminated (C-PDMS, lower structure), rendering hydrophobic IONPs, (**B**) Functionalization with PAA:PDMS-co-APMS, PAA coating followed attractive electrostatic interaction of PDMS-co-APMS with PAA coating (**C**). TEM image of IONPs produced in argon (later coated in C-PDMS) demonstrated cubic morphology (**D**), TEM image of IONPs produced in air (later coated with PAA:PDMS-co-APMS or PDMS-OH).

The IONPs produced under argon were coated with Siliclad or C-PDMS and the IONPs produced in air were coated with PAA:PDMS-co-APMS or PDMS-OH. IONPs emerge from synthesis coated in oleate. Oleate was removed by addition of HCl dropwise, which protonates the carboxyl group forming oleic acid [21,22]. IONPs are redispersed in chloroform (or a green alternative such as dimethoxyethane) for coating with PDMS-OH, Siliclad^®^, or C-PDMS (see Figure 1a). A large excess of each these polymers (3× by volume) was combined with IONPs followed by washing and centrifugation to remove free polymer. PAA:PDMS-co-APMS application was performed following a procedure published elsewhere [23] (see Figure 1b), in consideration of colloidal dispersion and polymer layering studies published elsewhere [24,25,26].

For this functionalization, 1% solutions of PAA and IONPs were combined initially, followed by a dialysis purification step, then PAA:IONPs were combined in a 1% PDMS-*co*-APMS block copolymers solution. Precipitation of the cationic IONP dispersion by PAA was performed by mixing an acidic solution of PAA and the acidic nanoparticle dispersion at a 1% by weight concentration at a 2:1 weight ratio using an excess of (PAA). After elimination of the supernatant, the pH was be increased by addition of potassium hydroxide. The precipitate redispersed spontaneously, as the now water-soluble IONPs became coated with PAA-coated NPs. The NPs were then dialyzed against water in 10 kD membrane (Slyde-A-Lyzer, Thermo Scientific, pers. Comm., Waltham, MA, USA) to remove the unlinked PAA polymer chains. The IONPs were extracted from the aqueous phase to the organic phase with diethyl ether. PDMS-co-APMS with viscosities of 80–120 cSt and 80–200 cSt, corresponding to a final length of ~6.5 nm and 18.7 nm, respectively, were used [23]. The PDMS-*co*-APMS block copolymers were dissolved in ethyl ether at a weight fraction of 1% and the solution was added to the colloidal IONP solution, and the two phases were mixed gently at room temperature [23]. The pH of the aqueous phase was decreased to 5.5 by HCl addition dropwise. IONPs were then extracted to the organic layer. The diethyl ether was removed, dried onto MgSO_4_ to remove water, and filtered. Solvents were removed under reduced pressure.

### 2.4. Characterization of IONPs

The IONP samples were characterized by transmission electron microscopy (TEM) using the JEOL 1200 EX TEM (Boston, MA, USA); IONPs were characterized by X-ray diffraction (XRD) prior to coating; NP-polymeric complexes were characterized by Fourier transform infrared spectroscopy (FTIR), zeta potential, and dynamic light scattering (DLS). Absorption measurements were performed on a scanning UV-vis spectrophotometer (Shimadzu UV-1800, Kyoto, Japan; North America: Shimadzu Scientific Instruments, Columbia, MD, USA) functionalized IONP samples were dispersed in isopropanol and scanned from 325–1100 nm. Magnetization measurements were performed on each sample using a Quantum Design MPMS XL superconducting quantum interference device (SQUID) magnetometer (Quantum Design North America, San Diego, CA, USA). We performed a field sweep at room temperature and a temperature sweep at 10 Oe.

### 2.5. Determination of Hydrophobicity via Contact Angle Measurements

Glass wafers were prepared by first cleaning with piranha etch (H_2_O_2_: H_2_SO_4_ at 1:3 *v*/*v*) at 60 °C for 20 min then washed thoroughly with deionized water and dried with nitrogen in the cleanroom. Prior to use, the glass wafers were washed sequentially with (1) acetone, (2) methanol, and (3) isopropoanol, rinsed again with DI water, and dried with nitrogen gas. The functionalized nanoparticles were dried to powder and dispersed in just enough isopropoanol to solvate them. The solutions were applied to the surface of the glass wafers and dried at 65 °C for two days to evaporate the isopropanol and fix the nanoparticles onto the glass surfaces. At that time, the static water contact angle was to be determined by gently placing one droplet (5 μL) of DI water onto the functionalized surfaces, one at a time, and photographing each droplet. Surface topography was determined with the SEM and surface plots were rendered using ImageJ, version 1.53k (Wayne Rasaband and contributors, National Institutes of Health, Bethesda, MD, USA). Contact angles were measured using the contact angle plugin on ImageJ.

### 2.6. Interaction and Magnetization of Plastic Nurdles and Fibers

To visually observe the IONPs adsorbing onto the surfaces of microplastics, polyethylene nurdles and polyethylene fibers (from 1000 multi-filament yarn, d = 30 µm) were used as models. Nurdles or fibers were dispersed in freshwater and IONPs solution was added to the 20 mL vials containing water and nurdles or fibers. Similarly, we performed preliminary binding studies using environmental nurdle samples collected from Packery channel, Corpus Christi University, TX (27°37.486′ N 97°12.883′ W), since environmental samples may have different surface properties than the new plastic beads with pristine surfaces. For this experiment approximately four drops of 30% *w*/*v* IONP solution was added to the petri dish or vial containing the environmental sample in 20 mL of water with a glass Pasteur pipette and mixed briefly with a glass stir rod.

### 2.7. Nanoparticle Removal from Water

Unfunctionalized PS NPs were combined with IONP and removed from water. Agglomerates of IONP-PS NPs were imaged using the JEOL Neoscope JCM-5000 benchtop SEM. To quantify binding, fluorescently labeled polystyrene NPs (Sigma-Aldrich, St. Louis, MO, USA) were diluted to 10 ppm with distilled water and combined with IONPs with PDMS-coating. The amphiphilic PAA:PDMS-co-APMS particles were not used in this case due to their strong positive zeta potential; we wanted to avoid observing electrostatic interactions between the fluorescent beads and the IONPs. The IONPs were added to the suspension and the mixture was incubated at room temperature overnight on a shaker operated at 200 rpm in a dark environment. The NP-water suspension without IONPs was used as control. The fluorescent signals of both IONP treated and control suspensions were measured on a BioTek Cytation 5 plate reader (BioTek Co., Winooski, VT, USA). For each NP, a standard curve of particle concentration vs. fluorescent signal intensities was produced with known concentrations of the NP. The concentrations of NPs, with and without IONP treatment, were calculated with the equations generated from the standard curves.

## 3. Results

### 3.1. TEM Characterization of IONPs

**Figure 2:**
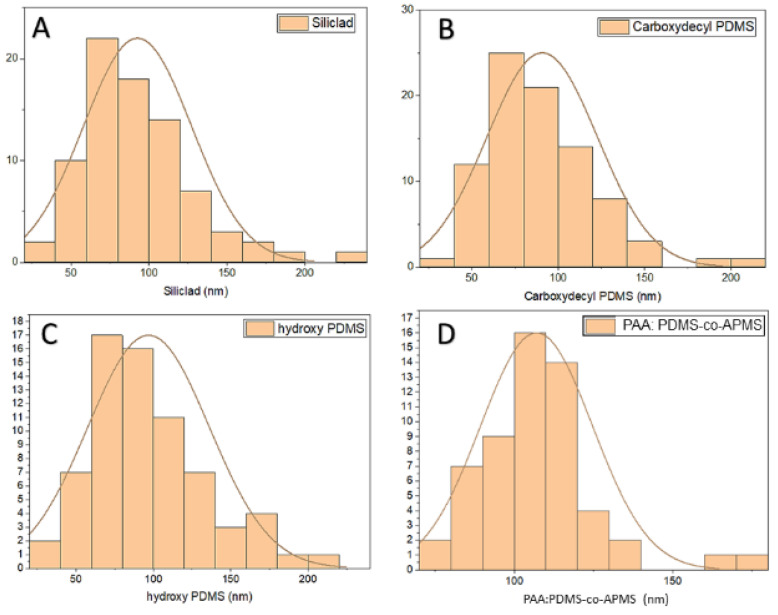
Size distribution histogram for IONPs coated with Siliclad (**A**). C-PDMS (**B**), PDMS-OH (**C**), and PAA:PDMS-co-APMS (**D**), line represents geometric mean.

The TEM images revealed a general cubic morphology. The sizes of the particles were as follows: Siliclad 92.65 nm (±34.73 nm), PAA:PDMS-co-APMS 107.02 nm (±17.69 nm), PDMS-OH 96.89 nm (±39.22 nm), and C-PDMS 90.79 nm (±32.01 nm). The size ranges were consistent whether the particles were produced under argon (Figure 1C) or in air (Figure 1D). Size distribution histograms from the TEM images are presented in Figure 2.

### 3.2. X-ray Diffraction

**Figure 3:**
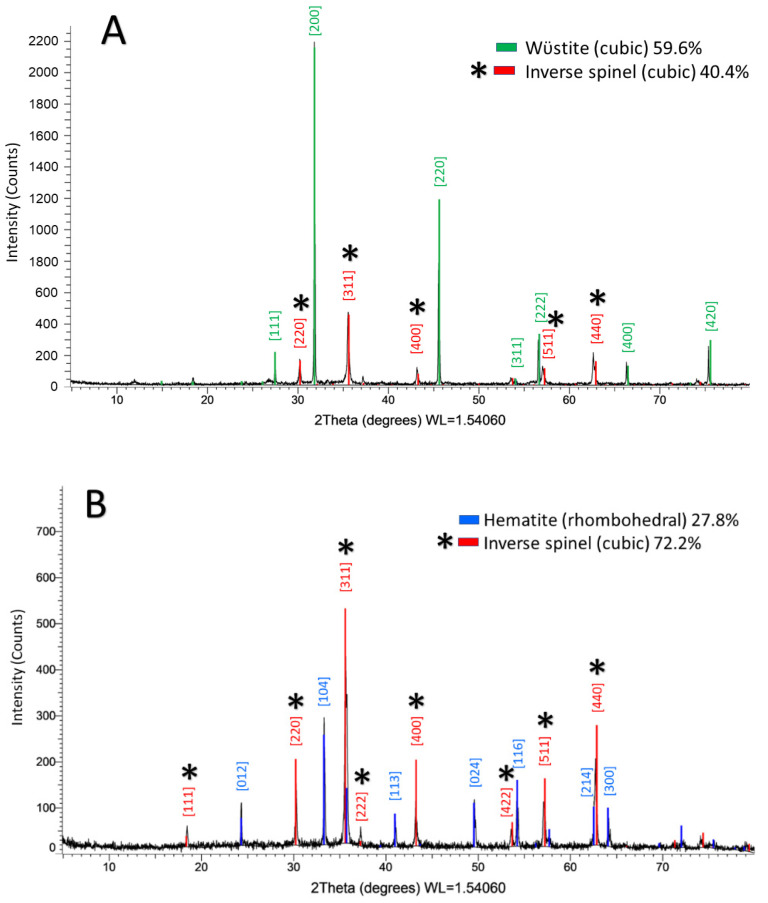
XRD spectra for IONPs. (**A**) XRD spectrum of IONPs produced using air-free techniques, under argon flow shows that the crystal phase is 59.6% wüstite and 40.4% *spinel phase (corresponds to starred indices in image), and (**B**) XRD spectrum of IONPs produced in ambient air shows that the crystal structure is 72.7% *spinel and 27.8% hematite.

XRD (θ/2θ) was performed on IONPs produced under argon or in air, prior to polymer functionalization, using a CuK_α_ source (λ = 1.54 nm). The XRD profiles of nanoparticles synthesized under argon flow (S1) and nanoparticles synthesized in ambient air (S2) both revealed two distinct phases. The inverse spinel phase(s) of iron oxide, which can be attributed either to the metastable phase maghemite (γ-Fe_2_O_3_) phase or the mixed valence phase magnetite (Fe_3_O_4_) [27] was present in both samples. The difference between these two inverse spinel phases could not be determined with certainty using XRD [21,28,29]. The spinel phase accounted for 40.4% (Figure 3A) of S1 and 72.2% (Figure 3B) of S2. S1 is also comprised of the wüstite (Fe_1−x_O) phase, space group Fm3m, 59.6% (Figure 3A) and S2 is also comprised of 27.8% (Figure 3B) rhombohedral hematite (α-Fe_2_O_3_) space group R-c3 (Figure 3B). By observing the XRD patterns alone, we would anticipate that S2 would perform better under magnetization measurements due to the higher percentage of crystal phase(s) with ferromagnetic ordering. Both the wüstite and α-Fe_2_O_3_ phases have antiferromagnetic ordering.

### 3.3. Zeta Potential

The amphiphilic composites PAA:PDMS-co-APMS were +65.49 mV, due to the presence of the amine groups on the co-block polymer, and hydrophobicity was confirmed for the PDMS-OH and the C-PDMS particles, which measured −3.82 mV, and −1.94, respectively, in DI water.

### 3.4. Dynamic Light Scattering

**Figure 4:**
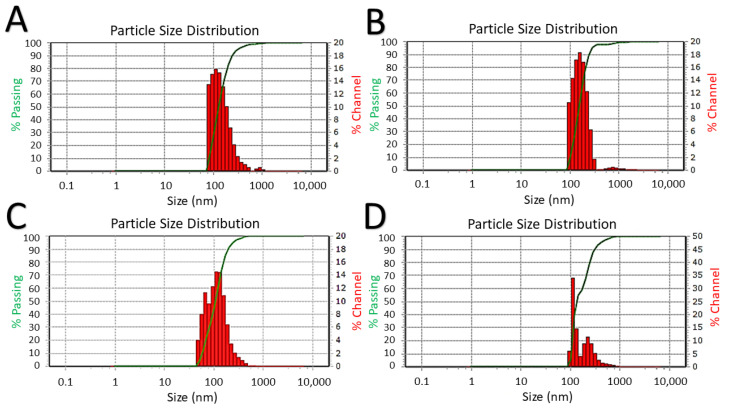
DLS size distribution histograms in water. Line represents cumulative size total. C-PDMS IONPs with SDS (**A**), PDMS-OH with SDS (**B**), PAA-coated (**C**), and PAA:PDMS-co-APMS IONPs (**D**).

Due to the poor colloidal stability of the hydrophobic PDMS-OH and the C-PDMS particles in water, it was necessary to combine them with SDS to render them soluble in water. The average hydrodynamic size of the C-PDMS@SDS was 160.1 nm (SD ± 60.70 nm, PDI 0.2835); the average hydrodynamic size of the PDMS-OH@SDS in water was 181.2 nm (SD ± 56.70 nm, PDI 0.1490); the average hydrodynamic size of PAA:PDMS-co-APMS IONPs was 186.5 nm (SD ± 78.40 nm, PDI 0.1809). DLS hydrodynamic size distribution histograms are summarized in Figure 4.

### 3.5. Magnetic Characterization

**Figure 5:**
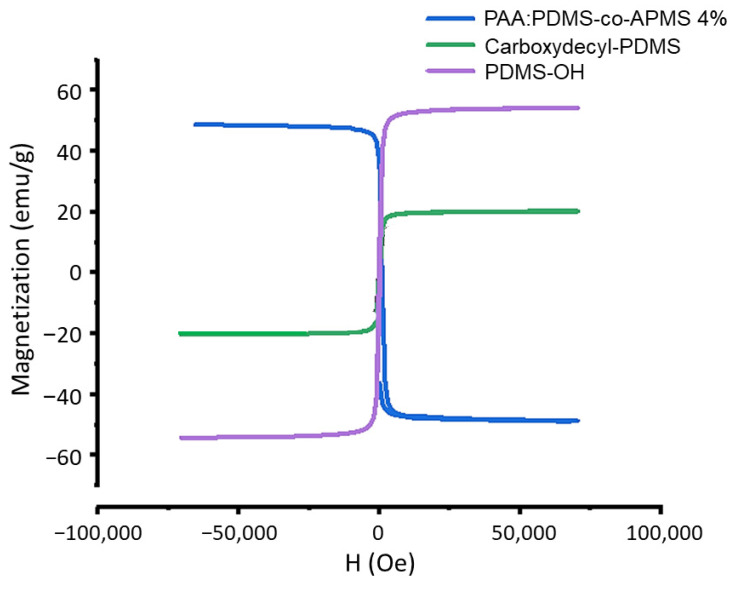
SQUID magnetometry hysteresis results, showing no coercivity and demonstrating the range of m_sat_ values for three different types of PDMS coated IONP samples; PAA:PDMS-co-APMS (4%) blue, carboxydecyl-PDMS green, and PDMS-OH purple.

As anticipated by the XRD findings, the particles produced in air have higher m_sat_ values (50–55 emu/g) than the IONPs produced under argon (20 emu/g), which we attribute to the higher percentage of the spinel phase, which has ferromagnetic ordering. The m_sat_ of bulk γ-Fe_2_O_3_ is 76 emu/g and the IONPs produced in air are closer to this value. The coating does not appear to significantly alter the magnetization (Figure 5). Superparamagnetism was observed in the hysteresis measurements as evidenced by the lack of coercivity.

**Figure 6:**
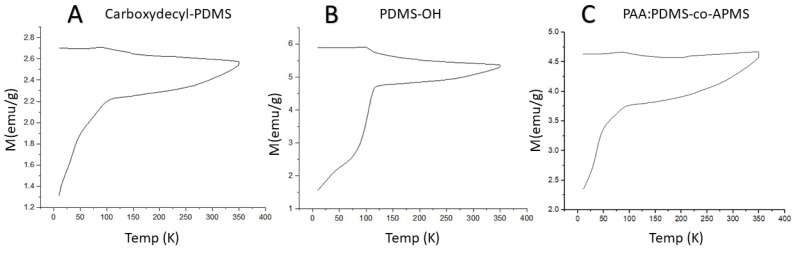
Zero-field cooled (ZFC, lower) and field-cooled (FC, upper) curves for the C-PDMS IONP sample (**A**); PDMS-OH IONP sample (**B**); and PAA:PDMS-co-APMS IONPs (**C**) taken at 10 Oe.

Field-cooled (FC) and zero-field cooled (ZFC) curves are presented in Figure 6A–C. We performed magnetization vs. temperature measurements of powder samples at temperatures from 4 to 350 K under a 10 Oe applied field (H). As observed in magnetic hysteresis measurements, the magnetization for the C-PDMS sample was lower than the other samples, with a maximum magnetization that was 55% lower than PDMS-OH and 43% lower than that of the PAA:PDMS-co-APMS sample. At the lowest temperature, the thermal energy of the dipoles in the IONPs will be at a minimum, as will the alignment with the external field, returning a small value for magnetization (M). With temperature increase, the thermal energy also increased, and the alignment of the dipoles could be facilitated, as evidenced by an increase in M up to the blocking temperature. The blocking temperature is generally regarded as the maximum of the ZFC curve. After the blocking temperature is reached, further temperature increases and increased thermal energy facilitate a decrease in the M value due to reduced dipole alignment with the field. We obtained the field cooled (FC) measurement by returning the temperature back to the starting temperature. Since these samples do not demonstrate any overlap between the FC and ZFC curves, we can conclude that there are significant dipole–dipole interactions, and a high degree of polydispersity, or a combination of both [30]. Below about 75 K and above 150 K the FC curve was nearly flat, but consistently demonstrated a slight, brief increase right around its maximum of 100 K. The ZFC experienced its most rapid increase in magnetization from 4 K through 100 K, after which it only increased slightly for the remainder of the temperature increase. The PAA:PDMS-co-APMS sample exhibited the largest increase in magnetization vs. temperature in the ZFC measurement above 100 K, as demonstrated by the slope of the line.

### 3.6. Fourier Transform Infrared Spectroscopy

**Figure 7:**
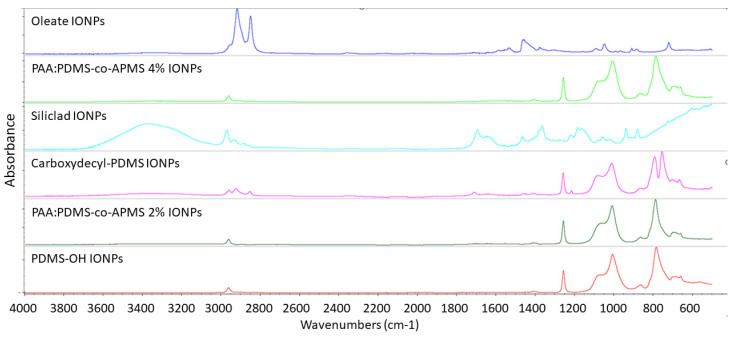
FTIR spectra for functionalized IONPs.

The polymeric fiber was a match to LDPE. The oleate-capped IONPs, directly out of synthesis, exhibit the two strong oleic acid peaks between 2800–3000 cm^−1^ corresponding to the CH_3_ and CH_2_-CH_2_ peaks which overlap close to 3000 cm^−1^ and the CH_2_-CH_3_ peak closer to 2800 cm^−1^. The functionalized IONPs (Figure 7) all returned a strong PDMS signature with PDMS being identified by FITR, with a >90% match to the library, even in the case of the multiple-polymeric IONPs. PDMS coated IONPs exhibited the characteristic PDMS IR peaks at 789–791 cm^−1^ due to the CH_3_ rocking (this signal was strongest in the C-PDMS sample) and Si–C stretch, 1020-1074 cm^−1^ which corresponded to Si–O–Si stretching, 1260–1259 cm^−1^ from the CH_3_ deformation of the Si-CH_3_, symmetric C–H bending at 1260 cm^−1^, and 2950–2960 cm^−1^ from the asymmetric stretch in Si–CH_3_.^SI3, SI4^ Si-C stretching can result in a peak at 690, 790 cm^−1^, [31,32]. C–H rocking around 843 cm^−1^, [32] asymmetric C–H bending at 1414 cm^−1^, present in all samples, but strongest in the C-PDMS, asymmetric C-H stretching at 2914–2965 cm^−1^, symmetric C–H stretching at 2847–2905 cm^−1^ Si–H stretching around 2158 cm^−1^ [31]. Many of the hydrocarbon peaks appeared stronger in the C-PDMS sample due to the long hydrocarbon chain it contains, the CH peaks between 2800–3000 cm^−1^, the methyl rock around 1200 cm^−1^, and the long-chain methyl rock at 700 cm^−1^ were clearly evident in this sample and lacking in others, as expected. The peak around 1700–1730 cm^−1^ corresponded to the carboxylic group of C-PDMS (decyl-COOH) [33,34] and did not appear in the other spectra. The bump in the area of 1600–1700 cm^−1^ in the PAA:PDMS-co-APMS samples can likely be attributed to the carboxyl group of PAA and/or the amide carbonyl group of APMS [35]. The signal from the secondary amine in the APMS is typically found at 3400 cm^1^, is typically a weak signal, and in this case was too weak to identify by FTIR. Peaks around 577 and 630 correspond to the Fe-O from the iron oxide particles. The small bump at 1631 cm^−1^ and around 3400 cm^−1^ were attributed to adsorbed water and surface hydroxyl groups bending and stretching, respectively [36].

### 3.7. UV-Vis Spectrophotometry

**Figure 8:**
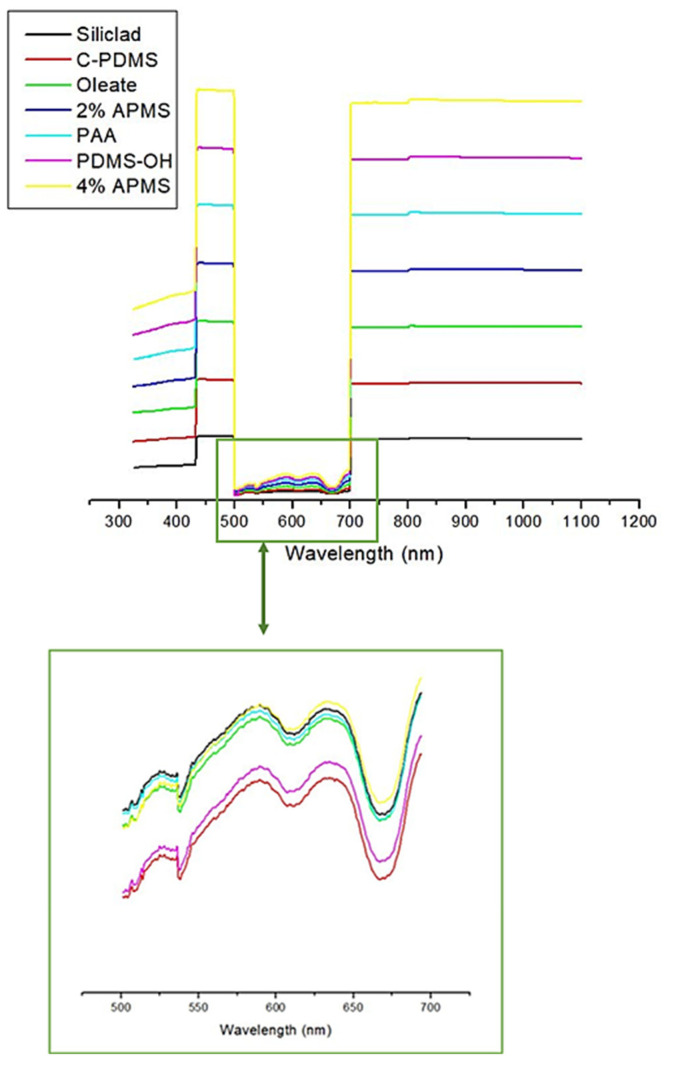
UV-vis absorption spectra for all IONP samples (**upper** image, full scan) and close-up of absorption in the visible range between 500–700 nm (**lower** image).

The iron oxide absorption appeared to dominate the spectra from 325–1100 nm. Strong absorption was observed in the ranges between 325 and 500 nm, and from 700–1100 nm, with significantly reduced, but persistent absorption in the wavelengths from 500–700 nm. No differences in the spectra for the differently functionalized IONP samples were observable using this technique (Figure 8). We also provide a scaled image of the spectra in the range from 500–700 nm, and absorption in this range is also identical for all samples (Figure 8, lower image).

### 3.8. Contact Angle Measurement Results

**Figure 9:**
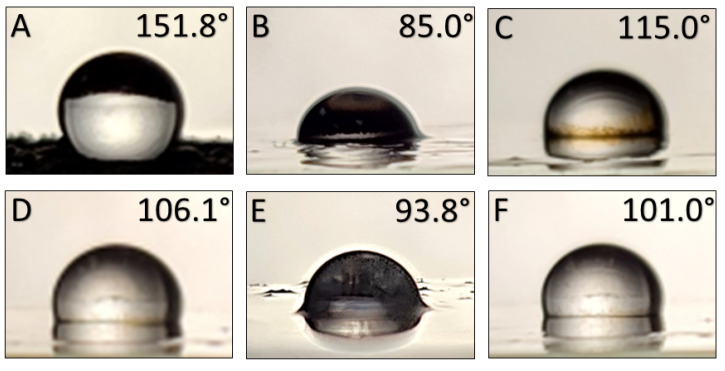
Static water contact angles, θ_c_, measured for all functionalized IONPs on glass substrates. Images for IONPS coated with (**A**) Siliclad, (**B**) hydroxy-PDMS, (**C**) oleate, (**D**) PAA:PDMS-co-APMS 4%, (**E**) PAA:PDMS-co-APMS 2%, and (**F**) carboxydecyl-PDMS.

**Figure 10:**
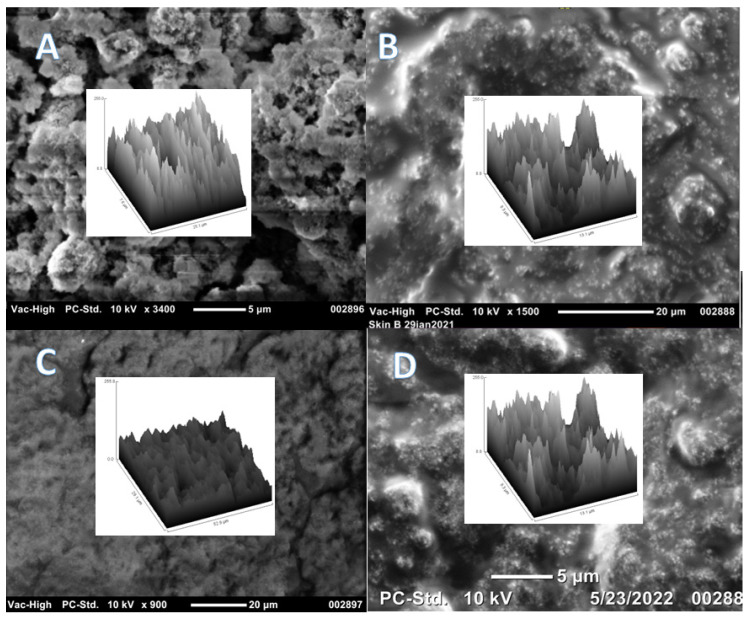
Scanning electron microscope (SEM) images showing topography of functionalized IONPs on glass wafers and surface plots (inlayed) for (**A**) Siliclad, (**B**) hydroxy-PDMS, PDMS-OH, (**C**) Oleate, and (**D**) PAA:PDMS-co-APMS 4%.

The images of the water droplets on the functionalized surfaces are provided in Figure 8; surface plots and SEM images of the four rough surfaces are presented in Figure 9. No topography for surfaces functionalized with either the 2% PAA:PDMS-co-APMS nor the carboxydecyl-PDMS IONPs was visible on the SEM despite the layer of reddish black IONPs being clearly visible with the naked eye. This suggests either a smooth surface, or topographical features below the minimum resolution of the SEM. The static water contact angle (θ_c_) for the functionalized nanoparticles on glass substrates was determined to be 151.8° for the Siliclad IONPs, 85.0° for the hydroxy-PDMS IONPs, 115.0° for the oleate coated IONPs, 106.1° for the 4% PAA:PDMS-co-APMS IONPs, 93.8° for the 2% PAA:PDMS-co-APMS IONPs; and 101.0° for the carboxydecyl-PDMS IONPs (see Figure 9). It is generally accepted that a static water contact angle θ_c_ > 90° is hydrophobic and θ_c_ < 90° is hydrophilic, however these conventions have been questioned [37]. It is interesting to note that a cutoff value of exactly 90° does not make sense physically, and it may be more accurate to consider hydrophobicity with regards to contact angle as a gradient, as opposed to a hard cutoff value [37]. Additionally, the surface roughness of the 4% PAA:PDMS-co-APMS, PDMS-OH, oleate, and Siliclad coated IONPs (Figure 10) likely affected these contact angle values [38]. More work is needed to determine why the topography was different among the samples, but interactions with the isopropanol solvent and drying effects are probable factors. Although the contact angle was small, and the surface roughness was observable for the PDMS-OH-coated IONPs, they clearly exhibited hydrophobic behavior in solution. It is possible that the PDMS-OH IONPs did not reach that cutoff value for hydrophobicity due to incomplete coverage of the IONP by the PDMS. More characterization studies are necessary to determine the degree of polymer coverage. The most hydrophobic functionalized IONPs we investigated appeared to be the Siliclad coated IONPs, reaching superhydrophobicity with a θ_c_ > 150° despite the surface roughness being comparable to the 4% PAA:PDMS-co-APMS. This type of functionalized IONP may be of interest for other surface functionalization applications. Interestingly, the amphiphilic polymers (PAA:PDMS-co-APMS) were also found to be hydrophobic, despite the strong charge and the presence of water-soluble functional groups. Although we would have anticipated that the polymer with the higher amine content (4%) would have exhibited a higher degree of hydrophilicity, that was not the case, rather the 2% was found to have the smaller θ_c_. This difference could possibly be attributed to overall polymer coverage onto the IONPs since identical IONPs were used for both procedures and the functionalization was performed in parallel. The amine content also affected the final polymer thickness on the IONP as reported previously [23], which may affect wettability.

### 3.9. Interactions with Plastic Particles and Fibers

**Figure 11:**
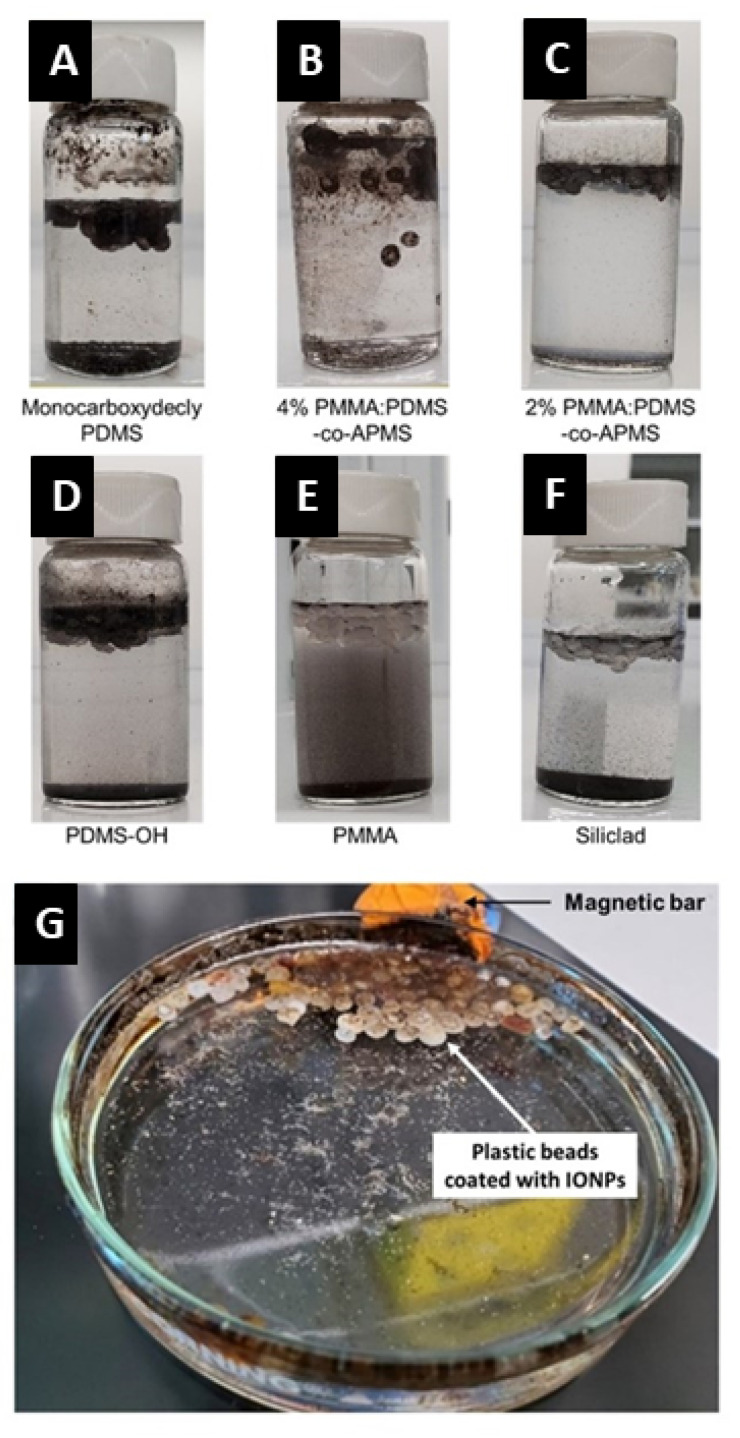
Attachment of IONPs to the nurdle samples collected from the environment. The attraction of environmental nurdles to the IONPs coated with C-PDMS. (**A**), 4% PAA:PDMS-co-APMS (**B**), 2% PAA:PDMS-co-APMS (**C**), PDMS-OH (**D**), PMMA (**E**), and Siliclad (**F**), was tested. The nurdles covered with the IONPs were collected by magnetic bars (**G**).

The best coverage and magnetization of the polyethylene nurdles was achieved by the C-PDMS (Figure 11A) and PAA:PDMS-co-APMS (Figure 11B,C) coated nanoparticles, followed by the PDMS-OH (Figure 11D). Binding was not observed for PAA alone (Figure 11E) or Siliclad^®^ (Figure 11F) coated IONPs, and PAA: PDMS-co-APMS binding was greater for the co-block polymer with 4% than 2% amine composition. However, the 4% had a strong affinity for the glass vial, possibly due to electrostatic interactions of the amino groups (Figure 11B,C). Siliclad^®^ particles were not further characterized due to poor performance in this experiment. We were also able to verify binding to the polyethylene fiber with SEM, as well as recovery of the polyethylene fibers. The interactions were tested in both freshwater and artificial sea water. Nurdles and fibers were visually inspected for binding of NPs and magnetically removed with static magnetic field (NdFeB) bar magnet with 100% recovery.

We were able to recover 100% of the environmental and pristine nurdles with a small 2” NdFeB magnet in both fresh and saltwater. Within minutes of adding the IONPs, the plastic particles were magnetized and a small 2” NdFeB magnet was used to instantly separate the plastic particles. The sand contained in the sample was not coated or removed and can be observed at the bottom of the petri dish in Figure 11G. Additionally, the sand, bentonite clay, and biologics left behind were not magnetized.

### 3.10. Binding to PS NPs

**Figure 12:**
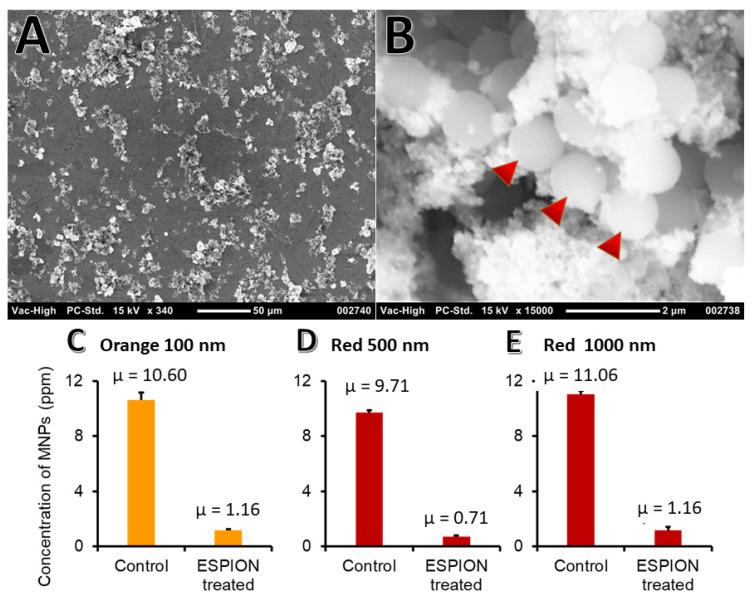
Binding of NPs by IONPs. After incubation with the IONPs, NPs in water were precipitated to form agglomerates (**A**). The NPs (arrows) and numerous IONPs (**B**). The removal of NPs with 100 (**C**), 500 (**D**), and 1000 (**E**) nm was tested using NPs with fluorescent tags.

Upon addition of the IONP solution, agglomerates were formed. The average agglomerate size obtained for the 1 µm PS beads was 50 µm (Figure 12A), a size that will facilitate more rapid filtration. SEM imaging was performed to view the interaction between the PS beads and the IONPs (Figure 12B). Enhanced separation could be performed with a magnetic laboratory filter combination. For the fluorescently labeled NMPs, after binding and recovery using a static magnet, the fluorescence of the supernatant was compared to the initial value and demonstrated a highly statistically significant reduction (*p* < 0.0001) in fluorescence. This corresponds to recoveries of NPs with 100 nm, 500 nm, and 1 μm approximately 89.1% (Figure 12C), 92.7% (Figure 12D), and 89.5% (Figure 12E), respectively.

## 4. Discussion

We have synthesized hydrophobic IONPs with various PDMS-functionalizations, including an amphiphilic co-block polymer. We have demonstrated binding and 100% recovery of naïve nurdle particles, as well as those exposed to environmental conditions in both freshwater and saltwater, and 90–93% recovery of nanoscale polystyrene in natural sea water using PDMS-functionalized IONPs to plastic particles. Although the PAA:PDMS-co-APMS functionalized IONPs were ideal candidates for this application due to their amphiphilic characteristics, the positive zeta potential may result in binding to biological moieties in undigested samples. However, this can be overcome by digesting biologicals prior to treatment, such as acid digestion, [39] strong base, [40,41,42] or enzymatic digestion [43,44]. The commercial PS beads, functionalized by surface carboxyl groups, are an ideal model for environmental samples with biologicals, such as an eco-corona on their surfaces, due to their strong negative zeta potential. However, the mechanisms of surface alteration of environmental nanoparticle samples by digestion methods are still unclear.

We have also demonstrated the ability to produce high m_sat_ IONPs in larger sizes (~100 nm) that can easily be removed using a simple permanent bar magnet. Better magnetic properties were observed for IONPs produced under ambient air than under argon flow (air-free), which we attribute to a higher percentage of the magnetic spinel phase of iron oxide in the nanocrystal structure. This is an interesting finding, considering the prevalence of air-free synthesis procedures. Synthesis in air significantly reduces complexity and costs.

Although we have used bar magnets to remove plastic particles from water samples in this study, and static magnetic fields appear sufficient for the separation of small volumes, for environmental remediation and wastewater purification, a high throughput system using HGMS is desirable due to the large volumes of water that would need to be processed. A multi-stage system, consisting of an electromagnet with on/off capabilities and a sonicator would be ideal for performing the necessary steps for binding, separation, and recycling of IONPs. Recycling capabilities would further enhance the sustainability of this approach.

IONPs with hydrophobic or amphiphilic coatings are a feasible option for the removal of NMPs in water, however, further research and development is necessary to optimize this system for environmental and water remediation. Iron oxide nanoparticles are ideal candidates for water remediation and the removal of a range of compounds of interest, including nanoplastics, via adsorption. IONPs are an environmentally friendly, cost-effective option. However, more work is needed to characterize the interparticle interactions and compare the laboratory models with environmental samples, optimize magnetic field types and strengths, and find sustainable methods for dealing with the micro and nanoplastics once they are collected. Plastics production does not appear to be slowing down, thus modern science must develop feasible methods for protecting the environment from plastic pollution fallout as we move toward a sustainable future.

## Data Availability

The data presented in this study are available on request from the corresponding author.
